# Damage-Free Shortening of Telomeres Is a Potential Strategy Supporting Blind Mole-Rat Longevity

**DOI:** 10.3390/genes14040845

**Published:** 2023-03-31

**Authors:** Huda Adwan Shekhidem, Lital Sharvit, Derek M. Huffman, Irena Manov, Gil Atzmon, Imad Shams

**Affiliations:** 1 Institute of Evolution, University of Haifa, Haifa 3498838, Israel; 2 Department of Evolutionary and Environmental Biology, University of Haifa, Haifa 3498838, Israel; 3 Department of Human Biology, University of Haifa, Haifa 3498838, Israel; 4 Departments of Molecular Pharmacology, Medicine, and the Institute for Aging Research, Albert Einstein College of Medicine, Bronx, NY 10461, USA

**Keywords:** *Spalax*, shelterin, telomere length, telomerase activity, senescence, DNA damage

## Abstract

Telomere shortening or loss of shelterin components activates DNA damage response (DDR) pathways, leading to a replicative senescence that is usually coupled with a senescence-associated secretory phenotype (SASP). Recent studies suggested that telomere aberration that activates DDR may occur, irrespective of telomere length or loss of shelterin complex. The blind mole-rat (*Spalax*) is a subterranean rodent with exceptional longevity, and its cells demonstrate an uncoupling of senescence and SASP inflammatory components. Herein, we evaluated *Spalax* relative telomere length, telomerase activity, and shelterin expression, along with telomere-associated DNA damage foci (TAFs) levels with cell passage. We show that telomeres shorten in *Spalax* fibroblasts similar to the process in rats, and that the telomerase activity is lower. Moreover, we found lower DNA damage foci at the telomeres and a decline in the mRNA expression of two shelterin proteins, known as ATM/ATR repressors. Although additional studies are required for understanding the underling mechanism, our present results imply that *Spalax* genome protection strategies include effective telomere maintenance, preventing early cellular senescence induced by persistent DDR, thereby contributing to its longevity and healthy aging.

## 1. Introduction

Significant efforts have been made towards the understanding of telomeres’ role in cellular senescence, aging, and age-associated diseases [1,2,3,4]. Telomeres are repetitive DNA sequences coated by the shelterin complex and located at the tips of eukaryotic chromosomes. Together, they shield the exposed ends and thereby, the genes located at the subtelomeric region, providing chromosomal stability by preventing their recognition as double strand breaks (DSB) and the activation of the DNA damage response (DDR) [5]. Telomeres can bypass the “end-replication” complexity caused by the incapability of the conventional polymerases to fully replicate the lagging DNA strand by recruiting a reverse transcriptase, known as telomerase, or by alternative mechanisms [6,7,8,9].

Since telomerase expression is restricted in most somatic cells, telomeric DNA progressively shortens with cell division [10]. As telomeres become shorter, they lose the ability to recruit sufficient shelterin components, which results in the exposure to DDR machinery, eventually leading to cellular senescence [11]. Recent studies have suggested that telomere aberrations triggering DDR can occur, irrespective of telomere length or the loss of the shelterin components (reviewed in [12]), indicating additional mechanisms inducing early senescence. Indeed, we have recently demonstrated shortening of telomeres in the blind mole-rat, of the genus *Nannospalax* (hereafter, *Spalax*), despite its long lifespan [13] and healthy aging processes [14], indicating a crucial role of telomeres’ integrity maintenance, rather than its elongation.

The senescent phenotype is usually accompanied by the senescence-associated secretory phenotype (SASP) that entails pro-inflammatory cytokines, as well as growth factors and extracellular matrix degrading proteins [15,16,17,18]. The chronic presence of senescent cells and a persistent SASP causes local and systemic inflammation that contributes to the development of age-associated diseases, which reinforce the role of cellular senescence in both aging and cancer. Nonetheless, senescence is associated with beneficial aspects, including tissue repair and wound healing, as well as embryonic development (reviewed in [19]). Hence, cell senescence is thought to contribute to keeping organisms relatively free from cancer in early life, but to possibly promote aging and age-associated pathologies in an antagonistic-pleiotropic manner later in life.

Throughout millions of years of underground evolution, *Spalax* has evolved superior survival mechanisms under hypoxia, which eventually promote cancer resistance and healthy aging [20,21,22]. Strikingly, the uncoupling of senescence and the SASP inflammatory response was recently discovered in *Spalax* [14]. *Spalax* is a solitary rodent that exhibits exceptional longevity (~20 years [23]), despite its small body size. Subterranean rodents naturally face great challenges due to repeated exposure to acute hypoxia and to internally-produced DNA-damaging substances that are released during hypoxia-reoxygenation cycles [24], yet *Spalax* seem to avoid such deleterious effects by efficient DNA repair mechanisms that protect cells from damage and subsequent oncogenic processes [25]. Notwithstanding these findings, limited data is available about *Spalax* telomere maintenance and telomerase activity and their contribution to cellular senescence in this wild animal. We have recently demonstrated that telomeres shorten with age in different *Spalax* tissues, similar to the process observed in short lived animals [13]; however, a brain transcriptome study showed that genes involved in telomere maintenance and associated with cellular resistance to DNA damage and telomere length regulation, such as *MRE11A*, *RNASEH*, and *TELO2*, were upregulated [26].

Here, we hypothesize that DNA integrity maintenance, including telomere regions, rather than telomere length maintenance, is a strategy that evolved in *Spalax* to support its exceptional healthy aging and longevity. To test this hypothesis, we employed primary fibroblast isolated from *Spalax* and laboratory rats in a cell culture model and measured telomere length, telomerase activity, telomere associated damage, and the expression of the shelterin complex mRNA.

## 2. Materials and Methods

### 2.1. Cell Culture

Blind mole-rat and laboratory rat (*Rattus norvegicus*) primary cultured fibroblast cells were tested. Cells were used for the evaluation of telomere length, telomerase activity, shelterin complex gene expression, and immuno-fluorescence analyses. Cells were isolated from three different newborn individuals (males and females, at an estimated age of 2–3 days), as described elsewhere [27]. The cell samples are listed in Supplementary Table S1.

The primary cell isolation protocol was approved by the Institutional Ethics Committee of the University of Haifa (reference #671/19).

The fibroblasts were grown in DMEM–F12 medium (supplemented with 10% FBS, L-glutamine (2 mM), and penicillin–streptomycin (100 U/mL, 0.1 mg/mL, respectively)) in a standard CO_2_ incubator. Growth media and supplements were purchased from Biological Industries (Beit HaEmek, Israel). In this study, we used young (second passage, P2), or senescent (fifth passage, P5) cells. Passage was recorded when a confluent culture was sub-cultured into 3 plates of the same area. Senescence was determined when the cells acquired an enlarged, flattened morphology and showed positive staining for senescence-associated β-galactosidase (Supplementary Figure S1). Other senescence markers (e.g., p21, p53, cell population doubling) were well described in previous studies of primary fibroblasts [14,25].

### 2.2. DNA Extraction

DNA was extracted from *Spalax* and rat primary cells using a High Pure PCR Template Preparation Kit (Roche, Penzberg, Germany), following the manufacturer’s protocol. All DNA samples were tested for purity and integrity using a Nanophotometer NP80 (Implen, Munchen, Germany).

### 2.3. Telomere Measurement

The average relative telomere length in primary cells was measured using qPCR (quantitative polymerase chain reaction) on a LightCycler 480 II (Roche, Penzberg, Germany), as previously described [28,29], and modified for use in *Spalax* and rats. The relative telomere length (T/S) of the samples was calculated as the ratio of the telomere concentration (T) to the single copy gene (S), relative to the reference sample standard curve. The erythropoietin (EPO) gene was used as a single copy gene for *Spalax* [30] and rats. The primers are listed in Supplementary Table S2. Both reactions for telomeres and single copy genes were run on the same plate in duplicates for each sample, along with a negative control of water. All reactions used 10 ng of DNA in a final volume of 20 µL containing 10 µL SYBER green Master Mix, 2 µL primers, 6 µL water, and 2 µL DNA sample. The reaction components and the LightCycler program are detailed in the Supplementary Tables S3 and S4.

### 2.4. RNA Extraction and Real-Time Quantitative PCR

RNA was extracted from cultured cells by using TRI Reagent (Molecular Research Center, Cincinnati, OH, USA), following the manufacturer’s instructions. The RNA samples were quantified on a Nanodrop^®^ spectrophotometer, and quality was assessed on the Agilent Bioanalyzer. RNA samples were treated with DNase I (DNA-free, Ambion, Austin, TX, USA), and 1 µg was taken for first-strand cDNA synthesis (iScript, Bio-Rad, Hercules, CA, USA) in a 20 μL volume. Aliquots of 1 μL of cDNA were used for each real-time PCR reaction. Species-specific primers were designed for each shelterin component transcript and telomerase using Primer3 software (Applied BioSystems, Austin, TX, USA), based on the published sequences (Supplementary Tables S5 and S6). Relative quantification of gene transcription was performed by using Fast SYBR Green (Applied BioSystems) and 1 µL of cDNA generated from 50 ng total RNA. Serial dilutions of the cDNA with the highest expression level for each target gene were used to build a relative standard curve and to test the amplification efficiency for each experiment. Samples were tested in triplicate. The amplification parameters were as follows: 95 °C for 5 min, followed by 45 cycles of 95 °C for 10 s, 60 °C for 10 s, and 75 ° C for 10 s. To verify a single product with a fixed melting temperature, the melting curve protocol was applied. The quantification relied on equal amounts of total RNA used in each sample, and the reliability of this method was tested and confirmed by cyclophilin and actin housekeeping genes (HKG) for *Spalax* and rats. The data are presented as relative gene expression values normalized to total RNA.

### 2.5. Telomerase Activity

Telomerase activity was measured in cultured cells, according to the telomeric repeat amplification protocol (TRAP) method [31], a sensitive and specific PCR-based functional enzyme assay. The assay was performed using the TeloTAGGG telomerase PCR ELISA PLUS (Roche Diagnostics GmbH, Singapore), following the manufacturer’s instructions.

Telomerase activity was measured according to the telomeric repeat amplification protocol (TRAP).

Briefly, each pellet from *Spalax* and rat primary cells was homogenized in 200 μL of lysis reagent. After a 30 min incubation on ice, the lysate was centrifuged at 16,000× *g* for 30 min at 4 °C, and the supernatant was aliquoted. Control templates, which contain positive telomerase template DNA with the same sequence as a telomerase product with eight telomeric repeats, were included in the kit. The PCR-based analysis was carried out in a 50 μL reaction mixture containing 3 μL cell extract. An internal standard was amplified by telomeric substrate primers in order to avoid false negative results. Telomerase positive results were confirmed by repeated TRAP assay, with heat pretreatment of cellular lysates (at 85 °C for 15 min) to monitor for false positive results. Lysis buffer reagent controls were included in each reaction to monitor for the possibility of reagent contamination. Positivity was detected when the substrate turned blue and then yellow upon the addition of the stop reagent, with the color conversion maximizing the sensitivity of the readings. Sample absorbance was measured within 30 min using a spectrophotometer at 450 nm against a blank (reference wavelength at 650 nm). Samples were considered as positive when the difference between the absorbance of the sample and the absorbance of the negative control was higher than the two-fold background activity. Telomerase activity was expressed as a ratio by comparing the signal of the sample to the signal of the high positive control template containing 0.1 μmol/μL DNA.

### 2.6. Immunofluorescence

Fibroblasts from late passages from both *Spalax* and rats were seeded in 6-well plates on glass coverslips at ~40 k cells/well and incubated overnight. The cells were then fixed with cold methanol for 10 min (−20 °C), permeabilized with 0.2% Triton X-100™ in PBS for 15 min, and blocked for one hour with 5% BSA solution in PBS. Staining: coverslips were incubated with the first primary antibody (anti γ-H2A.X, Abcam, Cambridge, UK) for 2  h at room temperature (diluted 1:700 in the staining blocking solution containing 0.05% triton and 1% BSA), washed three times, and incubated with the secondary antibody (goat anti-rabbit IgG Alexa fluor^®^ 488, Abcam, Cambridge, UK) in the dark for 1  h at room temperature (diluted 1:300 in the staining blocking solution), and washed three times with washing buffer containing 0.05% triton. The coverslips were then post-fixed with cold methanol for 10 min, air-dried, and stained with anti-TRF2 conjugated with Alexa fluor^®^ 594 (Novus Biologicals, CO, USA) at a concentration 1:200 in staining blocking solution and incubated in the dark overnight (4 °C). After a final washing, the coverslips were inverted onto slides containing Vectashield mounting medium and DAPI. The cells’ nuclei were visualized under a fluorescent microscope (Leica DMi8, equipped with Leica DFC365FX camera) and counted using Foci Counter software.

### 2.7. Statistical Analysis

All statistical analyses were performed by using JMP14 (SAS Institute Inc., Cary, NC, USA) and GraphPad Prism8 (San Diego, CA, USA). The correlation between rTL and age was calculated using Pearson’s correlation test. The lines on the graphs represent simple linear regression adjusted for age. Statistical comparisons were made using the Mann–Whitney U test for two groups.

## 3. Results

### 3.1. Relative Telomere Length and Telomerase Activity in Fibroblast Cells

**Figure 1:**
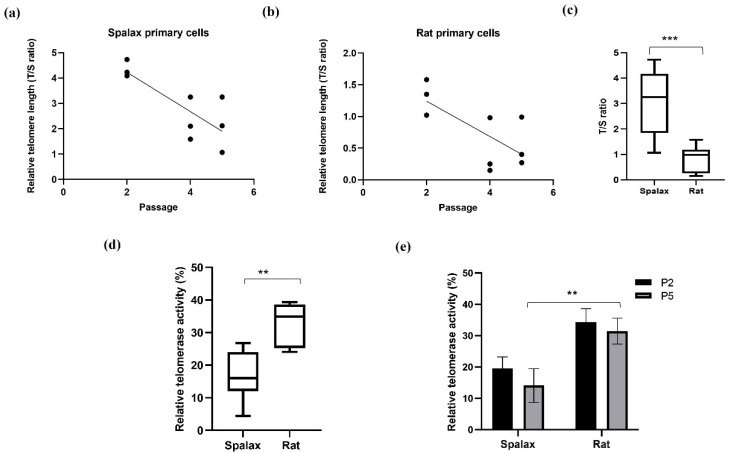
Relative telomere length (telomere to single copy gene (T/S) ratio) and relative telomerase activity in *Spalax* and rats’ primary fibroblasts. (**a**) Relative telomere length (rTL) as a function of passages in *Spalax* primary fibroblasts (slope = −0.7750, F1,7 = 12.6, *p* < 0.01, R^2^ = 0.6429); (**b**) relative telomere length (rTL) as a function of passages in rat primary fibroblast cells (slope = −0.2793, F1,7 = 6.969, *p* < 0.05, R^2^ = 0.4989) (Pearson’s); (**c**) range of rTL between *Spalax* and rat fibroblasts (boxplots represent data from **a**,**b**); (**d**) range of relative telomerase activity (rTA) between *Spalax* and rat fibroblasts (both passages); (**e**) rTA in *Spalax* and rat primary fibroblast cells with cell passage (*n* = 3, cells from three different individuals of *Spalax* and rats). ** *p* < 0.01 and *** *p* < 0.001 (Mann–Whitney U test).

We first analyzed the relative telomere length (rTL) and relative telomerase activity (rTA) in *Spalax* and rat fibroblasts from different passages (where the fifth passage demonstrated the senescence phenotype in both species). rTL in *Spalax* fibroblasts decreased with the cell passages (slope = −0.7750, F1,7 = 12.6, *p* < 0.01, R^2^ = 0.6429) (Figure 1a), similar to the results observed in short-lived rats (slope = −0.2793, F1,7 = 6.969, *p* < 0.05, R^2^ = 0.4989) (Figure 1b), and in line with our previous in vivo study [13]. Nonetheless, rTL was significantly higher in *Spalax* fibroblasts compared with those of rats (*p* < 0.0001) (Figure 1c), and both species showed a similar rTL decrease rate. We next tested telomerase activity (%) in lysates from primary fibroblasts. Overall rTA in *Spalax* was lower than in rats (Figure 1d), with no significant differences in the rTA between passage 2 and passage 5 (terminal passage for the primary cells) of the same species (Figure 1e). These results indicate a *Spalax*-specific strategy of long telomeres combined with inhibited telomerase activity.

### 3.2. Telomere Associated DNA Damage Foci (TAFs)

**Figure 2:**
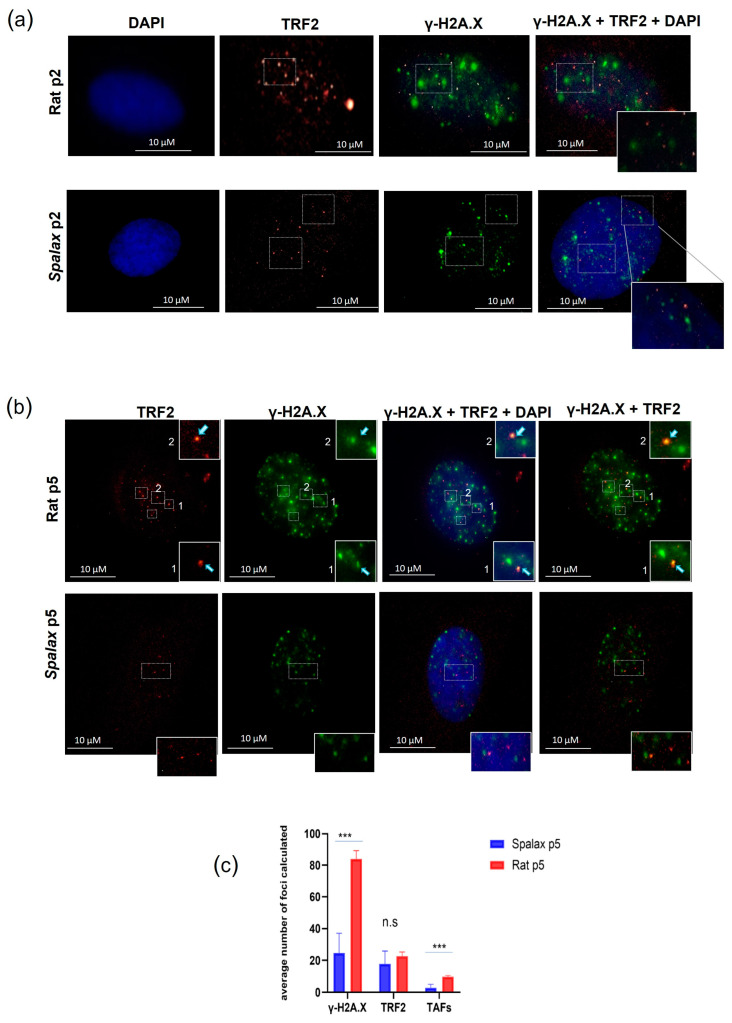
TAFs formation in *Spalax* and rat cells. Immunostaining for TRF2 (red) and γ-H2A.X (green) cell nuclei were counterstained with DAPI (blue) for early-passage cells (**a**) and late-passage, senescent cells (**b**). Examples of double stained foci are marked with rectangles. (**c**) The average number of γ-H2A.X, TRF2 and TAFs (co-localized) foci counted in 20 randomly chosen nuclei with γ-H2A.X positive foci using FociCounter. *** *p* < 0.001 (Mann–Whitney U-test).

Telomere associated foci (TAFs) are DNA damaged foci occurring in telomeres and can be induced as a result of the telomere shortening during replicative senescence or by the disruption of the shelterin complex. The uncapped telomeres recruit DDR factors, such as γ-H2A.X, and initiate signaling cascade, eventually leading to replicative senescence [32]. However, new studies show that damage in telomeres can occur in a length-independent manner [33]. Here, we investigated the accumulation of TAFs in senescent *Spalax* and rat fibroblasts by co-immunostaining for the DNA damage marker γ-H2A.X and the telomeric protein TRF2 (Figure 2). Our results are in line with previously reported higher general DNA damage in rat cells [14]. Indeed, senescent *Spalax* fibroblasts exhibited significantly less DNA damage in general than did those of the rats (Supplementary Figure S2) and specifically harbored less damage at the telomeric site (Figure 2a,b). While the average number of foci counted for the telomere marker did not differ significantly between rat and *Spalax* senescent fibroblasts (*p* = 0.1706) (Figure 2c), the average number counted for the colocalized markers γ-H2A.X and TRF2 was significantly higher in rats (*p* < 0.0001), indicating telomere-associated DNA damage. Conversely, the colocalization of TRF2 and γ-H2A.X among the nuclei of the *Spalax* senescent fibroblasts was almost absent.

### 3.3. Shelterin Core Components’ mRNA Expression Dynamics in Primary Fibroblast Cells

**Figure 3:**
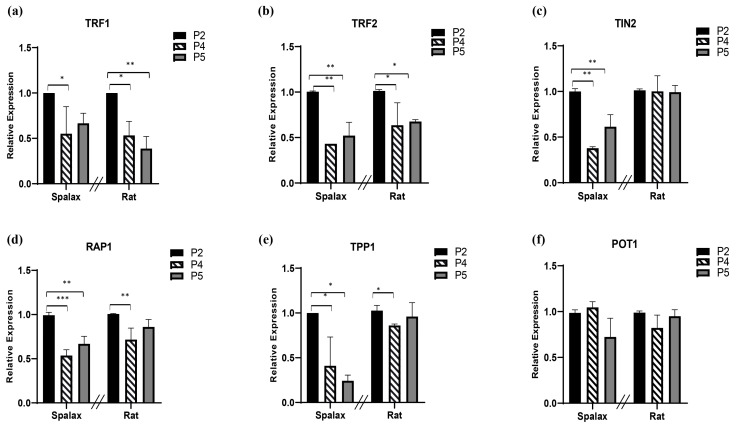
Levels of relative transcription of shelterin complex core components (**a**–**f**) in primary fibroblast cells of *Spalax* and rats. The mRNA expression rates were quantified in three different passages; passage 2, 4, and 5 (P2, P4 and P5) by using qRT–PCR. Relative transcription levels are presented as mean ± SD of three independent experiments (*n* = 3, cells from three different individuals of *Spalax* and rats). * *p* < 0.05, ** *p* < 0.01, and *** *p* < 0.001 (Mann–Whitney U test). Note that the relative standard curve was built for each species separately; therefore, data points of each species can only be compared to its control (1.0). The differences between P4 and P5 were insignificant.

Shelterin is essential for protecting telomeres from undesirable attack and binding of DNA damage response factors. The shelterin core proteins are listed in Table 1. Since loss of one component of this protection complex has detrimental effects on telomere function and stability [34], we next examined the relative RNA expression of these factors in vitro (Figure 3). TRF1 relative transcription declined with passages in both *Spalax* and rat cells (Figure 3a). The relative expression of both TRF2 and repressor/activator protein 1 RAP1 also decreased with passages in both animals, with more pronounced decline of RAP1 in *Spalax* cells (Figure 3b,d). TRF1 interacting nuclear protein 2 (TIN2) and TIN2-interacting protein-1 (TPP1) demonstrated a pronounced passage-dependent decline in transcription, mainly in *Spalax*. TIN2 showed stable transcription in rats along their life span, while TPP1 demonstrated a moderate decline in passage 4 rat cells; however, the expression level increased back to control levels (Figure 3c,e). Yet, protection of telomeres 1 (POT1) relative expression levels remained steady in both animals in all passages (Figure 3f). These results might indicate that the changes in telomere length and shelterin expression show similarities between the long-lived *Spalax* and the short-lived rat.

## 4. Discussion

In this study, we examined the average telomere length and telomerase activity, as well as the formation of telomere associated foci (TAFs) and the mRNA expression levels of the shelterin components in cultured primary cells of *Spalax*, a long-lived, hypoxia-tolerant, and cancer-resistant rodent. We showed that with cell passages, *Spalax* fibroblasts demonstrated significant shortening in telomere length, similar to rat cells, and in line with the processes observed earlier in tissues [13]. We also demonstrated that the average telomere length in *Spalax* fibroblasts was significantly higher than the average length in rats, similar to previously reported results in *Spalax* muscles [13]. Long telomeres are controversially described in the literature by their association with cancer risk, aging, or longevity. Extremely long telomeres in mice were reported to produce beneficial metabolic effects, low cancer risks, and longevity [35]. Whether the long, seemingly guarded telomeres are one of the driving forces in *Spalax* longevity and healthy aging remains unclear. It may be speculated that longer telomeres are attributed to telomerase overexpression, which presumably prolongs cell survival; however, we found that *Spalax* fibroblast telomerase activity was, in fact, lower than that of its counterpart in rats, which further supports our hypothesis that integrity maintenance of the telomeres, rather than telomere elongation, is characteristic of *Spalax* cells as a strategy that contributes to its long lifespan and supports its unique mode of cellular senescence. It was suggested that long-lived animals have adopted a mechanism [36] whereby the pace of telomere attrition and the activity of the telomerase is the same as that in other, short-lived animals. However, since initially, *Spalax* exhibits longer and potentially safeguarded telomeres, it seems tempting to speculate that the time it takes to reach critical length/damage that ignites the senescence machinery is longer and therefore, may contribute to their profoundly unique mode of replicative senescence lacking canonical inflammatory response known to accompany the senescent phenotype in all studied species. Therefore, replicative senescence in *Spalax* cells is seemingly not a direct consequence of telomere attrition and persistent DDR, but is rather determined by other intrinsic, underexplored mechanisms. Another tempting question is whether the downregulation of telomerase activity observed in *Spalax* cells plays a critical role in its cancer resistance.

Rapid and efficient DNA repair capacity was previously reported in *Spalax* cells in general [25], including low levels of double strand DNA break marker γ-H2A.X in *Spalax* senescent cells [14]. In accordance with these reports, here, we show lower telomeric DNA damage manifested as the extremely lower co-immunostaining of γ-H2A.X and telomeric marker TRF2 (Figure 2). This was not surprising, since less damage was shown in senescing *Spalax* cells [14], along with a higher expression of DNA repair genes [26] and a higher repair capacity of the cells [25]. Another aspect that contributes to genomic integrity in *Spalax* cells is the extreme abundance of the reduced glutathione antioxidant in *Spalax* cells under normoxia and hypoxia [37,38]. Taken together, efficient DNA repair and an enhanced antioxidant system may contribute to telomere integrity, thereby allowing the unique mode of senescence in *Spalax* cells.

We next addressed the complexes responsible for maintaining telomere integrity at the mRNA level (Table 1 and Figure 3) in order to test whether they are expressed differently in *Spalax*. Many studies have demonstrated that compromised shelterin function has deleterious consequences for the cell, and that telomeres have to bind sufficient amounts of shelterin in order to fully suppress DNA damage signaling and repair [11,39,40]. For instance, studies conducted on mice demonstrated that deletion of each shelterin component, with the exception of RAP1, leads to embryonic lethality (reviewed in [41]). In addition, several studies suggest that shelterin might modulate telomerase activity at the chromosome ends, mainly by acting as a negative regulator [42,43]. To our knowledge, this is the first study to examine shelterin expression levels with cell passages in *Spalax*. Cell passage-dependent changes in shelterin expression were similar in both *Spalax* and rat fibroblasts, apart from that of TIN2 and TPP1 (partially) commonly found bonded together, that decreased their levels with cell division in *Spalax*. Although there was a mild decrease in TPP1 transcription in the rat middle passage (passage 4), a further increase in passage 5 still indicates differences in the senescence phenotype between the two species.

TPP1 is known to elicit a robust damage response at the telomere ends upon deletion, and it is usually required for the recruitment of telomerase, acting as a positive regulator of telomere maintenance [44]. Yet, it may also be involved in telomerase negative regulation, together with POT1, but the precise molecular mechanism of this aspect is still not clear [45]. TIN2 is also a negative regulator of telomere length, where overexpression of TIN2 was found to inhibit telomere elongation [46]. More importantly, TIN2 binds TPP1 and is required for TPP1/POT1′s recruitment of the shelterin complex [47]. The TPP1/POT1 complex requires a connection to the TIN2/TRF1/TRF2 complex in order to repress the ATR DNA damage response. Another TIN2 function is to promote the repression of ATM signaling by TRF2. The fact that the expression levels of these two ATM/ATR repressors decreased with cell division in *Spalax* may partially support the enhanced activity of DNA repair in *Spalax* [25]. However, this suggestion requires verification and further studies, since high ATM/ATR activity requires better protection of the telomere ends; furthermore, one of the limitations of this study is that the mRNA expression data presented here may not necessarily represent protein levels; moreover, specific commercial antibodies for *Spalax* as a non-model organism are not always available, and if so, the identical specificity to the compared species is not guaranteed. This issue might be a subject for future studies.

In summary, our results support that *Spalax* have evolved strategies for genome protection that apparently include telomere maintenance machinery, together contributing to its longevity and healthy aging. These strategies include a unique mode of senescence not induced by persistent DDR or telomere attrition, but which rather seems to be an independent cell program driven by other types of ‘clocks’. The precise mechanisms of telomere maintenance and the apparently ‘non-canonical senescence clock’ require further investigation in *Spalax* and other long-lived species as possible requisites for long lifespan and healthy aging.

## Figures and Tables

**Table 1:** Shelterin components and function.

Protein	Full Name	Binds to:	Function	Expression-Change with Cell Passage	
				*Spalax*	Rat
TRF1	Telomere repeat binding factor 1	double-stranded telomeric DNA	telomerase repressor, negative regulator of telomere length	↓	↓
TRF2	Telomere repeat binding factor 2	double-stranded telomeric DNA	telomerase repressor, negative regulator of telomere length, T-loop formation and protection, repressor of ATM response	↓	↓
POT1	Protection of Telomeres 1	single-stranded telomeric overhang	activator/inhibitor of telomerase	-	-
TIN2	TRF1 interacting nuclear protein 2	TRF1 and TRF2	repressor of ATR and ATM signaling by linking the POT1-TPP1 heterodimer and stabilizing TRF at the telomeres	↓	-
TPP1	TIN2-interacting protein 1	POT1	recruitment of telomerase	↓	-
RAP1	Repressor/activator protein 1	TRF2	repressing the cleavage of the T-loop by preventing the activation of DDR factors	↓	↓

(↓) expression downregulated; (-) expression unchanged.

## Data Availability

The data that support the findings of this study are available in the Methods section and the Supplementary Material of this article.

## Supplementary Materials

The following supporting information can be downloaded at: https://www.mdpi.com/article/10.3390/genes14040845/s1, Figure S1: Senescence was determined when the cells acquired an enlarged, flattened morphology and showed positive staining for senescence-associated β-galactosidase; Figure S2: Levels of DNA damage in *Spalax* and rat fibroblasts; Table S1: List of animals and their ages; Table S2: List of primers for telomere length assay; Table S3: Reaction components; Table S4: PCR program; Table S5: List of primers for *Spalax* shelterin complex; Table S6: List of primers for rat shelterin complex.
