# Supplementary material for: Damage-Free Shortening of Telomeres Is a Potential Strategy Supporting Blind Mole-Rat Longevity
Source: Genes (Basel). 2023 Mar 31;14(4):845. doi: 10.3390/genes14040845 (PMC10137574; doi:10.3390/genes14040845)
Supplement: Supplementary file 1 [file genes-14-00845-s001.zip › genes-2256395-supplementary.pdf]

Damage-free shortening telomeres is a potential strategy supporting blind mole rat longevity

Online-Only Supplemental Material

Table S1: List of animals and their ages

Figure S1: β-galactosidase staining of senescent cells

Table S2: List of primers for telomere length assay

Table S3: Reaction components

Table S4: PCR program

Table S5: List of primers for Spalax shelterin complex

Table S6: List of primers for Rat shelterin complex

Figure S2: Levels of DNA damage in Spalax and rat fibroblasts

Table S1: List of animals and their ages

	Species	Age
Cells	***Spalax***	2 days
		2 days
		3 days
	**Rat**	3 days
		2 days
		3 days

Figure S1:

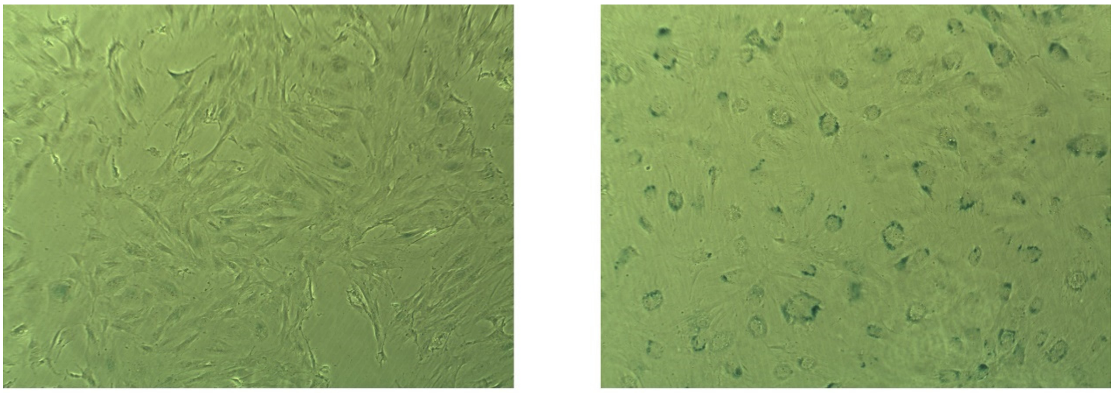

Young Spalax Fibroblasts p=2 Senescent Spalax Fibroblasts p=5

Figure S1: Senescence was determined when the cells acquired an enlarged, flattened morphology and showed positive staining for senescence-associated β-galactosidase.

Table S2: List of primers for telomere length assay

Primer	Forward sequence	Reverse sequence	Species
Telo	5'-CGGTTTGTTTGGGTTTGGGTTTGGGTTTGGGTTTGGGTT-3'	5'-GGCTTGCCTTACCCTTACCCTTACCCTTACCCTTACCCT-3'	All
EPO	5'- GGCTGTAGAGGTCTGGCAAGG-3'	5'-GGAATTGGCTAGCACAGCCT-3'	*Spalax*
EPO	5'-ACGCCTCATTTGCGACAGTC-3'	5'-GCCCATTGTGACATTTTCTGC-3'	Rat

Table S3: Reaction components

**Telo**	**Telo x1**	**SCG x1**
DDW	6µl	6µl
Primer F	0.2µl	0.6µl
Primer R	1.8µl	1.4µl
RXN mix	10µl	10µl
DNA or serial dilution	2µl	2µl

Table S4: PCR program

Initial Denaturation	95°C for 10 min	
Denaturation	95°C for 10 sec	×50
Annealing	60°C for 5 sec	
Synthesis	72°C for 11 sec	

Table S5: List of primers for *Spalax* shelterin complex

Primer	Forward sequence	Reverse sequence
TRF1	5'-TGGACTTCCTCTGCCTCTCT-3'	5': TAATAGCTTCCGCGCTGTCA-3'
TRF2	5'- ATCACCATCCAAGCCCACAG-3'	5'-GCTGTTCCACTTGCCTTTGG-3'
TPP1	5'-TGAGGTACAGGAGGACCAGG-3'	5'-GGGTAACAGAGGTGGGTGTG-3'
POT1	5'- ACTTGTCAGCTCTTGGGCAA-3'	5'-AAATGGTGTCCTGGTGCCAT-3'
TIN2	5'-AGTCTGCAGCGAGAAAACGA-3'	5'-AGGCCTCTGACAGTTGCTTC-3'
RAP1	5'- GGATACAGCAGGGACAGAGC-3'	5'-ACTAGTGCAAACCCTTGGCC-3'

Table S6: List of primers for Rat shelterin complex

Primer	Forward sequence	Reverse sequence
TRF1	5'- GACAGCGCCGAGGCTATTAT-'3	5'-TCCTGCTGCAACTCTTGTCA-'3
TRF2	5'-GACCAGCTGTTTGAAGTGCG-3'	5'- TCCTTCACCCACTCGCTTTC-3'
TPP1	5'- GAAGGACAACTCCCAGCCTC-3'	5'-CTGCAGGGTGTAGAACTGGG-3'
POT1	5'- TCGGGGGATTAGGGTTTTGC-3'	5'-TGACTGCCGAATCTGCACAT-3'
TIN2	5'-TGTGAAGCAGCTGTCAGAGG-3'	5'-TCCATGGCAACCAGAAAGGG-3'
RAP1	5`- TGCTGGAAATCCTGGACACC-3'	5'- TAGTGCAAACCCTTGGCCAT-3'

Figure S2:

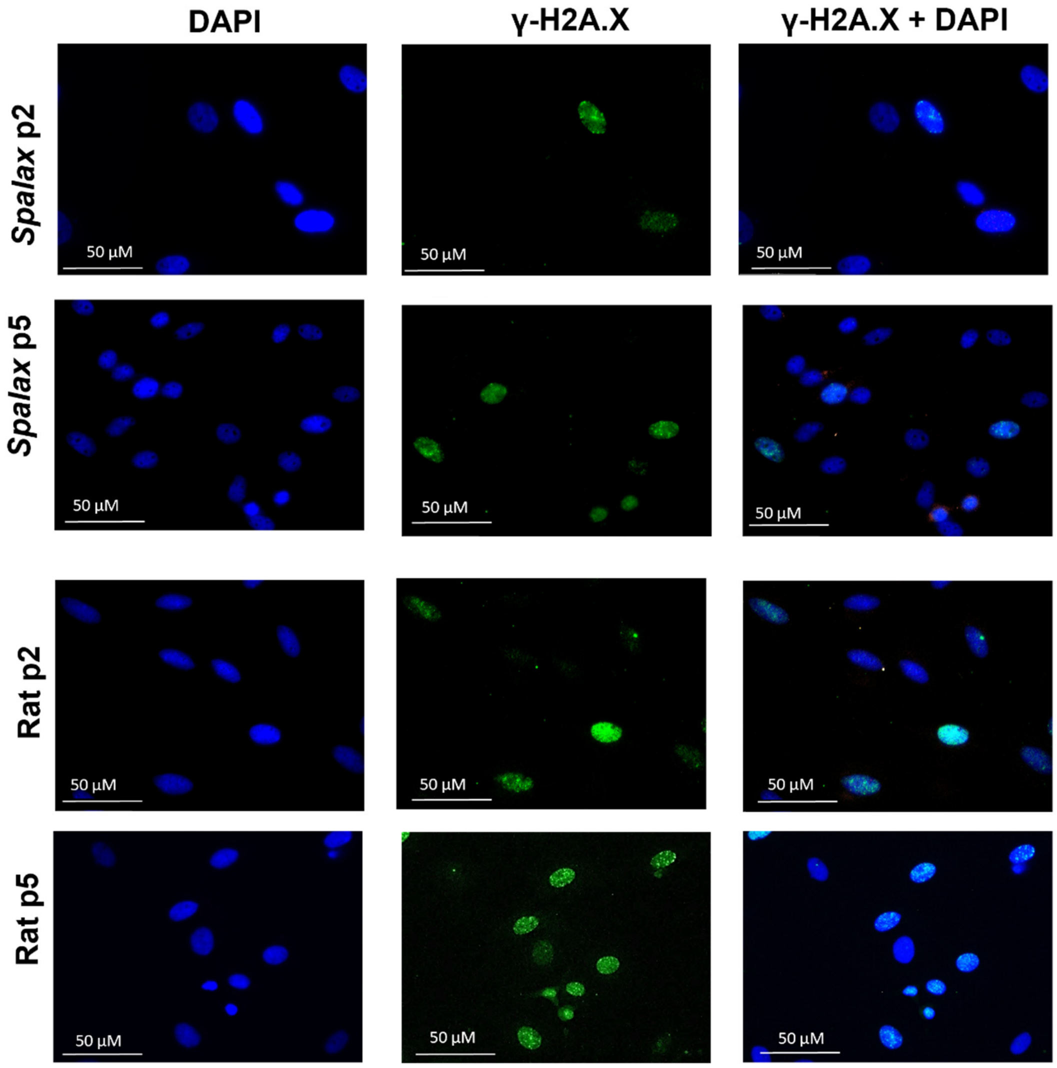

Figure S2: The levels of DNA damage in Spalax and rat fibroblasts (representative images).
